# Agent-based modeling of malaria vectors: the importance of spatial simulation

**DOI:** 10.1186/1756-3305-7-308

**Published:** 2014-07-03

**Authors:** Arne Bomblies

**Affiliations:** 1 University of Vermont, School of Engineering, 33 Colchester Ave, Burlington, VT 05405, USA

**Keywords:** Agent-based modeling, Malaria, Topography, *Anopheles gambiae*

## Abstract

**Background:**

The modeling of malaria vector mosquito populations yields great insight into drivers of malaria transmission at the village scale. Simulation of individual mosquitoes as “agents” in a distributed, dynamic model domain may be greatly beneficial for simulation of spatial relationships of vectors and hosts.

**Methods:**

In this study, an agent-based model is used to simulate the life cycle and movement of individual malaria vector mosquitoes in a Niger Sahel village, with individual simulated mosquitoes interacting with their physical environment as well as humans. Various processes that are known to be epidemiologically important, such as the dependence of parity on flight distance between developmental habitat and blood meal hosts and therefore spatial relationships of pools and houses, are readily simulated using this modeling paradigm. Impacts of perturbations can be evaluated on the basis of vectorial capacity, because the interactions between individuals that make up the population- scale metric vectorial capacity can be easily tracked for simulated mosquitoes and human blood meal hosts, without the need to estimate vectorial capacity parameters.

**Results:**

As expected, model results show pronounced impacts of pool source reduction from larvicide application and draining, but with varying degrees of impact depending on the spatial relationship between pools and human habitation. Results highlight the importance of spatially-explicit simulation that can model individuals such as in an agent-based model.

**Conclusions:**

The impacts of perturbations on village scale malaria transmission depend on spatial locations of individual mosquitoes, as well as the tracking of relevant life cycle events and characteristics of individual mosquitoes. This study demonstrates advantages of using an agent-based approach for village-scale mosquito simulation to address questions in which spatial relationships are known to be important.

## Background

Malaria transmission is highly complex, with transmission dynamics affected by drivers such as climate, weather, hydrology, social dynamics and the effects of the human immune system. All of these can simultaneously influence temporal and spatial patterns of malaria transmission. Consequently, the causal relationship between any one driver of malaria and human disease burden may not be immediately evident in many transmission situations.

Mathematical and numerical models of malaria transmission can yield illuminating insight about many aspects of the vector/host/parasite system by isolating the impact of certain cause-effect relationships and controlling for other variables. For example, models have been applied to assess the impacts of interventions such as insecticide-treated bednets e.g. [1,2], the impacts of climatic variability and change e.g. [3-5] and the impacts of acquired immunity e.g. [6-8]. Such models often rely on a compartmental structure in which a number of individual humans or mosquitoes transition from one categorization to the next at rates that may be fixed parameters or dependent on some external driver such as climate and internal model variables such as mosquito abundance. Differential equations form the basis of this model formulation, and the compartments represent states (for instance, “susceptible” for human hosts). The model compartments assume spatial homogeneity and uniform characteristics of individuals within each compartment. It is not possible to track characteristics of specific individuals; it is only possible to update the status of groups. Many variations of these models exist, and they have yielded many great insights into the transmission system. However, a very different model structure—the agent-based model—may add insight for studying certain epidemiologically important processes that are often ignored in compartmental models because their structure does not lend itself to simulation of these processes. For instance, the dependence of malaria transmission on spatial heterogeneity of standing water and human habitation is readily explored using spatially explicit, individual-based simulation but would be cumbersome in compartmental models. The known importance of this process in village-scale malaria transmission can be illustrated using an agent-based model by analyzing various scenarios in a well-known transmission environment. Such approaches to studying malaria transmission are rare, however, one notable agent-based modeling study assessed the proximity of source reduction to human habitation [9], in which the spatially-explicit nature of the agent-based model was leveraged to test various environmental management scenarios. Another agent-based model was developed by Bomblies *et al*. [10], and was coupled to a dynamic hydrology model. This modeling study builds on the previous applications of agent-based models to further demonstrate their utility in understanding various drivers and controls of malaria transmission.

Agent-based models (also known as individual-based models) simulate individuals as autonomous actors that behave according to a set of prescribed rules and can share information with each other. Individual agents do not behave according to a top-down governing structure. Rather, they are configured to behave autonomously in pursuit of personal goals, and population-scale behavior stems from agents’ collective actions for a bottom-up simulation structure. Simulated mosquitoes are not restricted to compartments, and are free to move in a model domain as they alternate between seeking blood meals and oviposition sites while interacting with their immediate environment. The simulated mosquito population can be visualized much like the actual population: as a cloud of points in the model domain representing individuals at their locations at particular times. The more commonly-applied compartmental models would lump mosquitoes into an aggregated number of individuals and lose the spatial information. In the agent-based model, relevant characteristics are tracked for individual mosquitoes, including location, age, infection and blood meal status, parous rate, and size. Each of these attributes are updated for each model time step as the mosquitoes move randomly throughout the model domain in search of oviposition habitat or blood meals depending on their stage in the life cycle.

Vectorial capacity is the daily rate of potential new infectious mosquito bites resulting from a blood meal taken from an infectious human. It is determined by the equation:

(1) $$\mathrm{VC}=\frac{ma^{2}p^{n}}{-\ln\left(p\right)}$$

where *m* = abundance term (mosquitoes per human); *a* = number of blood meals per vector per day; *p* = daily survival probability of vectors (0–1); *n* = extrinsic incubation period of parasite in days. The vectorial capacity is commonly calculated by parameterizing equation 1 using estimates of *a* and *m*, and calculating values of *p* and *n* using established functions of temperature. However, in an agent-based model, the number of blood meals and mosquito movement are tracked in the model for each individual, and therefore, the population-level transmission variable vectorial capacity can be determined by counting the vector-host interactions occurring in a simulation of human and mosquito agents and aggregating to the population level. In this way, model agent interactions determine vectorial capacity parameters and there is no need for error-prone parameter estimates. This study uses this simple vectorial capacity output to evaluate a variety of hypothetical transmission scenarios by simulating changes to the physical environment (such as pool drainage and larvicide application) and implications for malaria control.

Agent-based models maintain the spatial relationships of individual mosquitoes with human blood meal hosts and the pooled water that constitutes developmental habitat. For some modeling applications, the characteristic spatial scale of variability in mosquito populations is much smaller than the computational grid cell, and spatial homogeneity is a reasonable assumption. However, in some transmission environments, empirical evidence points to the importance of spatial heterogeneity of physical drivers such as village-scale hydrology and mosquito breeding habitat [10-13], spatially variable adult mosquito populations e.g. [13,14] and resulting malaria prevalence or vectorial capacity e.g. [13,15-19]. The flight distance between developmental habitat and human residences can be a significant risk factor [20]. Some compartmental models incorporate village scale spatial heterogeneity using “patches” of perfectly-mixed compartments to simulate spatial heterogeneity in oviposition sites, humans, and subsets of the mosquito population, and the resulting impacts on malaria transmission [20,21].

## Methods

Banizoumbou is a village of about 1000 inhabitants located in southwestern Niger (13° 31’ N, 2° 39’ E) in a semiarid plateau landscape of tiger bush, shrubland, and millet crops. The village is typical of the Sahel. A pronounced rainy season from May through October controls mosquito populations in this water-limited environment, and mosquito abundance is closely tied to rainfall. Several stream channels cross the landscape, but they are geomorphological relicts of a wetter period and most runoff collects in small topographic low points to form pools that are typically several meters to tens of meters in diameter. Stream channels almost never contain flowing water. When persistent enough to allow eggs that have been deposited in the pools to mature to adult mosquitoes, these pools facilitate expansion of the mosquito population in Banizoumbou. The interplay of rainfall patterns with topography can strongly influence pool persistence [10,22] and therefore topography can play a leading role in spatial heterogeneity of mosquito abundance within a village. Areas with more persistently pooled water see more mosquito activity than areas with short-duration puddles. All scenarios are evaluated using the model HYDREMATS [10], which loosely couples a distributed hydrology model to an agent-based mosquito population model. HYDREMATS simulates both human and mosquito agents. From June 2005 through 2008, Bomblies *et al.*[10] made observations in Banizoumbou to calibrate and validate HYDREMATS for that village. They determined that *Anopheles arabiensis* and *An gambiae sensu stricto* are the two dominant malaria vectors in Banizoumbou and collected weekly samples of mosquitoes at several locations to track temporal trends in abundance, and also measured the hydrological and meteorological characteristics at hourly time steps using automated data logging devices.

**Figure 1:**
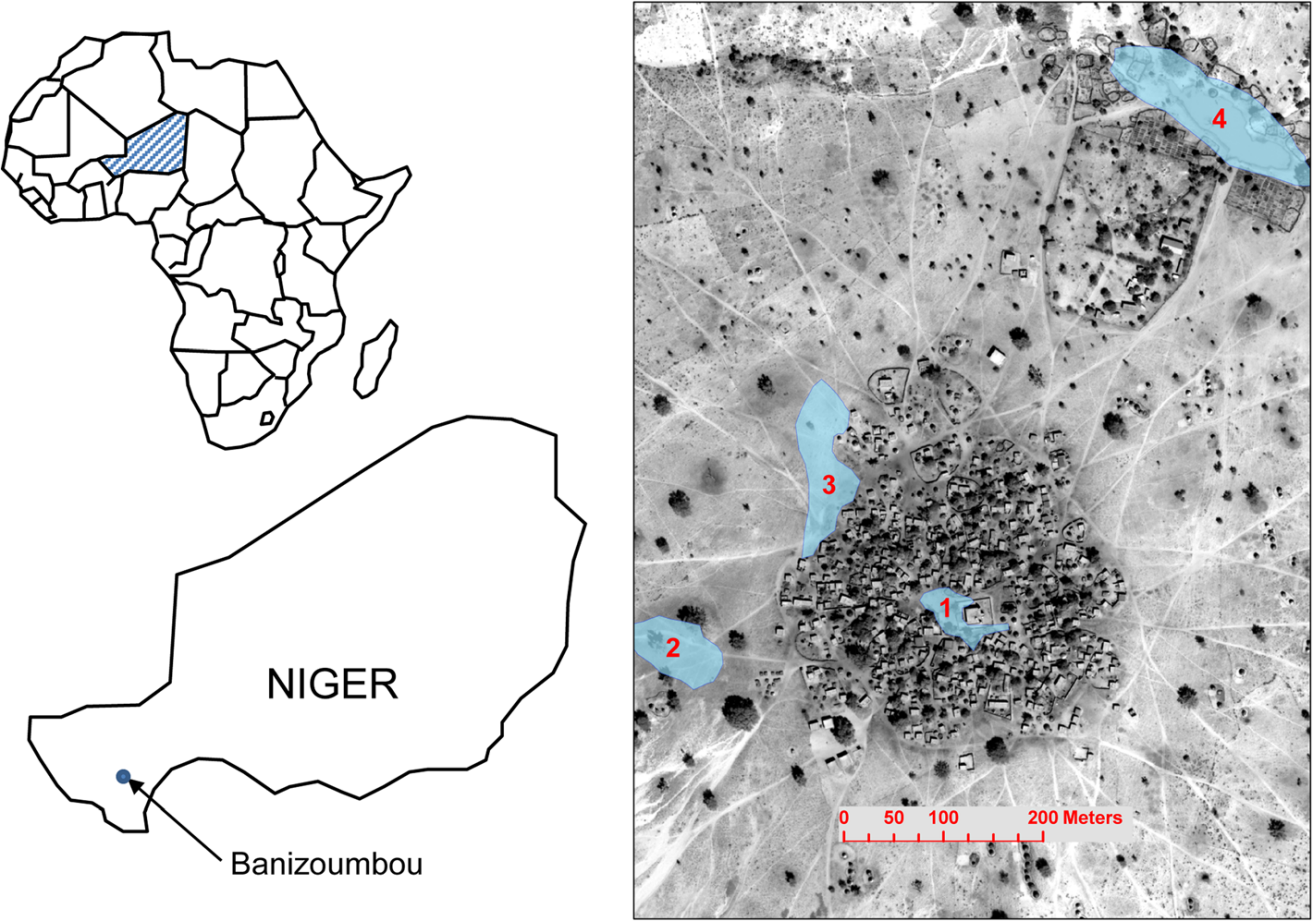
**Left panel: The location of Banizoumbou.** Right panel: a Quickbird satellite panchromatic image of Banizoumbou Village with recurrent, productive pools outlined as polygons. Other, smaller and less persistent pools formed in Banizoumbou and yielded adult mosquitoes, but are not shown on this figure.

The vectorial capacity is modeled by tracking the interactions of mosquito and human agents in the model. These can vary spatially depending on local mosquito density and the life stage of the particular mosquito (host-seeking, gravid, resting). When an initial blood meal is taken from a human in the model, the individual mosquito’s status is updated to track which human was the source of the blood meal. Next, the mosquito responds to simulated CO_2_ gradients to determine flight direction and developmental pool location as it cycles between taking a blood meal, resting, laying eggs, and seeking its next blood meal. These behaviors are well documented in the literature and details of the model are described in [10]. When an individual mosquito takes a blood meal from a human, the human blood meal host’s unique identification becomes part of the mosquito’s attributes that are tracked during the mosquito’s life. When the mosquito, during her life span, takes a second bite after the required period of sporogony (111 degree-days above 18°C are necessary for sporogony [23]), the initial host gets “credited” with a potential secondary infection, because the initial blood meal host’s identification and the number of bites are known for each individual mosquito. If the secondary bite precedes the requisite duration of sporogony, then the host is not credited with a secondary bite. The number of secondary infections is tracked for each human, and the average for all humans in the simulation is the population-level vectorial capacity at that moment in time. For comparison purposes, the vectorial capacity is then time-integrated. The time-integration of hourly vectorial capacity can be interpreted as a cumulative potential transmission impact of the vector population over the rainy season for the village.Because the development of individual mosquitoes occurs in pools, the mosquito abundance is tied to pool persistence, which must exceed the temperature-dependent maturation time of subadult mosquitoes. Moreover, mosquito abundance depends on the proximity of pools to houses, because gravid mosquitoes must find their way back to a pool for oviposition. The mosquito is less likely to encounter the pool if it is far away. Figure 1 shows the location of Banizoumbou, as well as a Quickbird satellite panchromatic image at 60 cm resolution. The location and maximum extent of the four largest, most persistent pools in the village are superimposed on the satellite image of the village (pools 1–4), but many other smaller pools that appear following rainfall are not shown in the figure. From field investigations in 2005, pools 1–4 were found to yield the most pupae (considered a proxy for adult mosquito eclosion), but smaller numbers of mosquitoes emerged from many less significant, smaller pools as well.

The hydrology model incorporates spatial variability in soil and vegetation type, as well as high resolution topography to make predictions of hourly soil moisture and pooled depths of water over the model domain. Evaporation and infiltration deplete runoff-generated water that has pooled in topographic low points, and if a specific pool’s persistence is less than the interstorm return period, then the aquatic-stage mosquitoes developing in that pool will not reach maturity and will not add to the village’s mosquito population. The model assumes that desiccation results in complete sterilization. In this manner, the interplay of rainfall variability (frequency, duration and intensity), hydrology, and topography lead to a spatially variable source of adult mosquitoes. Once emerged, mosquitoes fly in a weighted radial random walk (Brownian motion) in search of a blood meal, but a component of their flight direction is dictated by carbon dioxide gradients, as mentioned above. Because they result from partially random flight to encounter blood meal hosts, mosquito blood meals are usually—but not necessarily—taken near the mosquito’s breeding habitat, and the mosquito population and malaria risk therefore varies throughout the village as in other field studies e.g. [11]. The model is applied for 2005 in the village of Banizoumbou. Many measurements of adult mosquito abundance and hydrological variables were taken during that year in order to validate the model, and these are fully described in Bomblies *et al*. [10].

**Figure 2:**
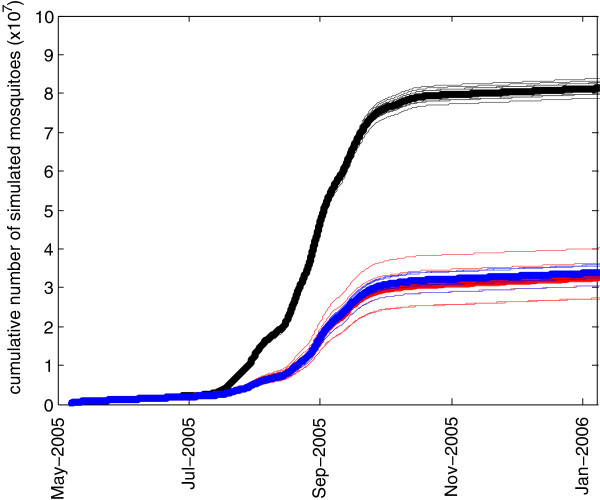
End of season time-integrated vectorial capacity for modeled scenarios, including source reduction for pools 1–4.

Several scenarios are explored using the model. First, the impact of source reduction to control mosquitoes is simulated for discrete individual pools. Two distinct methods are simulated by the model: first, it is assumed that pools are rendered unproductive by larvicide use. In the model, the eclosion of adult mosquitoes is prohibited from pools that have received larvicide applications, but mosquitoes can still use the standing water in the pool for oviposition. It is assumed that mosquitoes still see the pools as suitable breeding habitat, even though their offspring will die in the treated water. This is done sequentially for pools 1–4 in different simulations. Next, each pool (1–4) is removed from a new simulation to evaluate the impact of the draining of the standing water, thus preventing mosquitoes from ovipositing.HYDREMATS is a deterministic model, but because mosquito behavior is simulated as having random components, an analysis of impacts must account for variance due to internal model stochasticity arising from the use of random number generators. Individual mosquito decisions are based on uniformly-distributed random numbers that are compared to model parameters to simulate behavior. For each scenario, a total of ten model replicates using different random number seeds were made to analyze the range of deterministic model outcomes. These ten replicates are summarized by the box plots in Figure 2 for each scenario.

## Results

**Figure 3:**
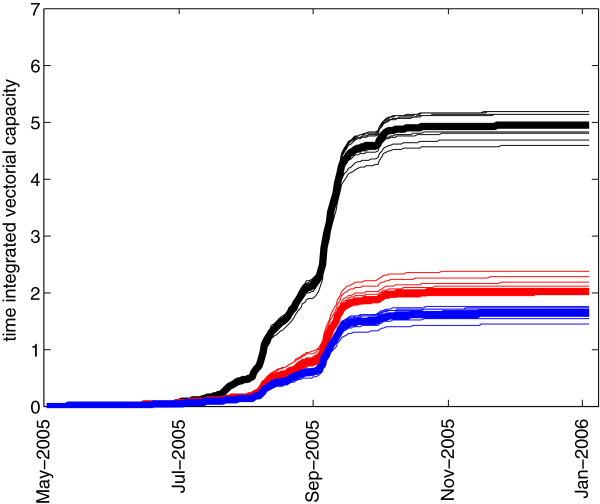
**Plot of time series of simulated cumulative mosquito abundance for the baseline 2005 scenario (black), as well as source reduction in Pool 1.** The scenarios of larvicide application (red) and pool draining (blue) are shown. Individual model replicates are thin lines, and the thick lines represent the average of ten replicates.

**Figure 4:**
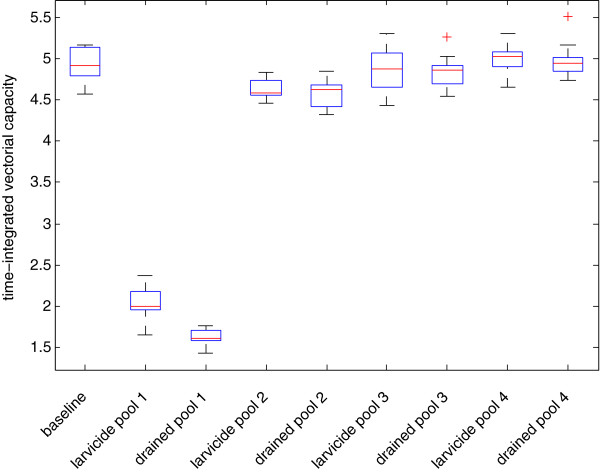
**Plot of time series of simulated time-integrated vectorial capacity for the baseline 2005 scenario (black), as well as source reduction in Pool 1.** The scenarios of larvicide application (red) and pool draining (blue) are shown. Individual model replicates are thin lines, and the thick lines represent the average of ten replicates.

Figure 3 shows the time series of simulated mosquito abundance for the 2005 baseline scenario (the same simulation as in [10]) and two perturbation scenarios for pool 1, which is located in the middle of the village: the application of larvicide and the draining of the pool. As seen in the figure, both scenarios that inhibit eclosion from pool 1 greatly reduce the total seasonal abundance, and the magnitude of the decreased abundance is approximately the same. In Figure 3, cumulative simulated mosquitoes are shown in order to smooth out short-term variability in populations, which can make the interpretation of a time series of abundance difficult. Approximately 46% of all of the simulated adult mosquitoes, over the entire season, eclose from pools 1–4. This fraction of mosquitoes having originated from pools 1–4 is about 80% during the early days of wetness, but falls back to a low of 35% after predators become established and density-dependent processes keep the numbers of emergent adults in check. In total over the season, 54% of all adult mosquitoes eclose from the smaller pools that dot the village. About 52% of the total vectorial capacity is due to mosquitoes from pools 1–4, indicating that this small subset of the large number of pools in the village contributes disproportionately to village malaria transmission. Upon removal of pool 1 in the simulation, the percentage drops to 29% of adults eclosing from pools 2–4. The difference in the apparent 60% reduction in total abundance resulting from the removal of pool 1 (Figure 3) and the 46% percentage of all mosquitoes from pools 1–4 is due to the nonlinear effects of pool 1: the mosquitoes that originated in pool 1 propagate in the model and some of them oviposit in pool 1, and some oviposit in other smaller transient pools. With the removal of pool 1, the offspring of pool 1 mosquitoes are absent from the simulation and the overall abundance is reduced, with fewer gravid mosquitoes to oviposit in any water body and with greater distance from blood meals compared to pool 1, the number of gravid mosquitoes is reduced. Thus, the productivity of other pools depends on the existence of the highly productive pool 1, and pool 1 is particularly important because of the proximity to human habitation. This nonlinear population effect is the reason for the apparent discrepancy.Figure 4 shows the simulated vectorial capacity for the same set of scenarios: baseline, pool 1 drained, and pool 1 with larvicide applied (i.e. the pool is available for oviposition, but no adult mosquitoes can eclose). Draining the pool, however, reduces the vectorial capacity more than the application of larvicide. As a female mosquito repeatedly cycles through taking a blood meal from a human host, resting, generating eggs (gonotrophy), oviposition, and again questing for a blood meal, she must fly certain distances between blood meal hosts and oviposition habitat. This greater flight distance takes time. If pools and blood meal sources are close together (such as in the case of pool 1), then the chance of a flying mosquito encountering the pool is much greater than if the nearest pool’s location is unknown and the mosquito must randomly fly until she encounters water. As a result, in a mosquito’s life span more secondary bites are possible if the time per cycle is shorter. This can be recognized in the agent-based simulation by tracking the average number of gonotrophic cycles per mosquito, for the entire population. For the baseline case, the maximum value attained by this metric was 0.904, compared to 0.875 for the larvicide scenario and only 0.696 following drainage of pool 1. Time-integrated values for this metric were 1918 for the baseline case, 2057 for the larvicide application scenario, and 1616 for the removal of the pool. Thus, this mechanism explains how the drainage of standing water from a centrally-located pool such as Pool 1 has a greater impact on reducing vectorial capacity than the application of larvicide. The importance of centrally located pool 1 as a dangerous source of mosquitoes may be obvious, but by tracking individuals the model is able to simulate mechanistically the population-scale nonlinear effects of spatial relations among individuals and features of the physical environment, which goes well beyond simply recognizing that a centrally-located pool is very dangerous.

**Figure 5:**
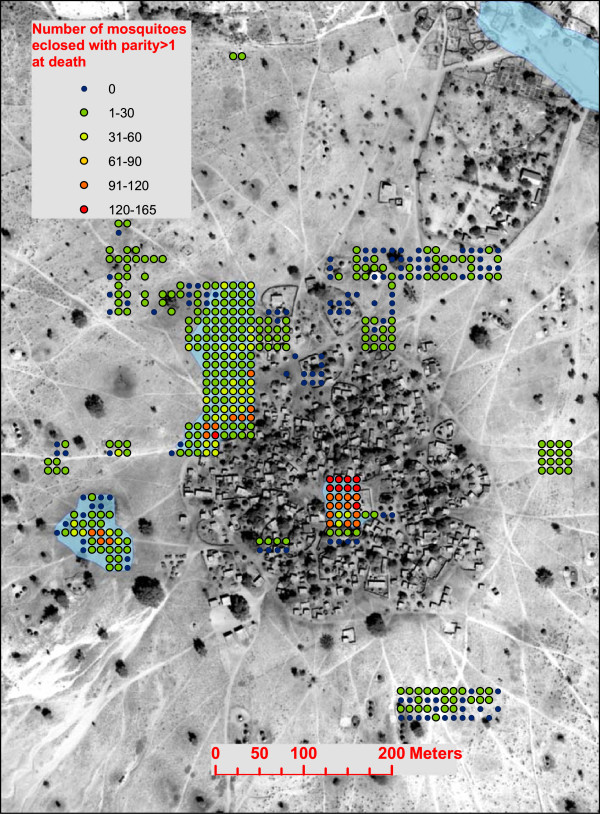
**The number of potentially dangerous (parity > 1) mosquitoes eclosing from modeled pools around the village.** The color scale shows the number of eclosed mosquitoes per computational grid cell.

Similar simulation experiments were conducted for pools 2, 3, and 4. The reductions in the 2005 season cumulative vectorial capacity are shown in Figure 2 for the removal of pools 2–4, and for the larvicide application on pools 2–4. Only the final cumulative vectorial capacity at the end of the season is shown in the figure in order to show the impacts of each of the perturbations. The results can be interpreted as the potential seasonal total number of secondary cases per initial case caused by the mosquito population. It is a time integration of the daily vectorial capacity, and is therefore no longer a rate but a seasonal total. Increases in this measure are related to population dynamics (more standing water leads to more mosquitoes), biting patterns arising from the spatial layout of pools and villages, and the age of mosquitoes (which allows more blood meals). Pools 2–4 are all located at the periphery of the village. For this reason, the impacts of pool elimination are not as pronounced for pools 2–4 as for pool 1 located in the center of the village. Mosquitoes that emerge from pools 2–4 are less likely to reproduce, because the mosquitoes will only find blood meal hosts in the direction of the village, and none if they fly in any other direction after eclosion. In contrast, mosquitoes eclosing from Pool 1 will find blood meal hosts if they fly in any direction. Livestock was not allowed to be near the village during the summer monsoon growing season, and therefore it is assumed that no animals exist in the simulation from which *An gambiae s.l* can draw blood meals. Pool 4, although it is the largest and most persistent water body in Banizoumbou, has the least impact on mosquito abundance and vectorial capacity because of its distance from the village.Simulated mosquitoes track and update their characteristics as they fly through the model domain. Each individual simulated mosquito retains memory of its site of eclosion. Figure 5 shows the locations of eclosion for all mosquitoes in the baseline scenario. The color gradient of markers shows the number of mosquitoes originating at each location that have parity greater than one at death, and thus contributed to the vectorial capacity. The most “dangerous” mosquitoes (the greatest number of gonotrophic cycles) emerge from pool 1, as expected.

## Discussion

Agent-based models are often applied to situations in which population-level behavior arises from the interactions of individuals. As such, they are well-suited for the simulation of vector-borne diseases such as malaria. For vector-borne diseases, vectorial capacity is a population-level metric that describes the collective potential of individual mosquitoes to transmit pathogens in host populations. This study has made use of the tracking of individual mosquitoes through their life cycles to simulate vectorial capacity for the entire population. Using this formulation, the impacts of changes to the physical environment (such as the removal of breeding habitat at various locations) are shown to be variably modeled, as a result of the spatially explicit structure of the model. This method is distinct from the more common approach of determining vectorial capacity from estimation of parameters, and evaluation of the sensitivity of those parameters to perturbations in the physical environment regardless of location of those changes. In contrast, agent-based models allow the changes to vectorial capacity to arise from individuals’ collective actions and interactions with spatially-distributed environmental features, interactions with humans, and their choice of breeding habitat.

Model results reproduce the known importance of spatial relationships of developmental habitat and human habitation by sequentially simulating the removal of several pools spaced at various distances from houses. Compartmentally-structured models do not have this capability. The danger to humans living near mosquito larval habitats is well known, yet many models assume perfect mixing of the mosquito population without regard to individual mosquito locations relative to developmental habitat or human habitation. The removal of pool 1 (located in the middle of the village) resulted in a much more dramatic decrease in both mosquito abundance and vectorial capacity than the removal of the other pools. The importance of pool 1 for the mosquito population was recognized in the field in 2005, and in 2007 that pool was the target of a larvicide application to assess the response in village mosquito abundance [24]. In that study, a 49% decrease in village adult mosquito abundance was observed following the application of the growth-inhibiting larvicide derived from crushed neem seeds to pool 1. The field-observed reduction in abundance is not as large as the modeled reduction of 63% following the application of larvicide, but the efficacy of the growth-inhibiting neem seeds used in that study was not perfect. Some pupae were observed in the pool, primarily because the neem powder was driven by wind to certain sections of the pool, leaving other sections of the pool untreated. The model assumes that no mosquitoes emerge from the pool following treatment. Nevertheless, the impact of the inhibition of mosquito eclosion from pool 1 is pronounced, both in observations and in the model results. The same cannot be said for the more peripheral pools studied (2–4). Model results show a significantly lower impact on the village-scale mosquito population as a result of the removal of these pools. The reduced impact is primarily because the mosquitoes that do emerge from these pools have a lower chance of finding suitable blood meal hosts because they are more distant and may not encounter villagers, and recently blood-fed mosquitoes seeking an oviposition site are less likely to encounter the more distant pools than the more proximal pool 1.

Different forms of environmental modifications can be simulated, and their impacts also depend on spatial relations. The impact of pool 1 elimination is noted in the reduction of vectorial capacity. However, the impact of draining the pool is greater than the impact of applying larvicide to the pool. Because the abundance does not differ between the draining and larvicide scenarios, the difference in vectorial capacity can be attributed to the differences in mosquito life cycle following each of the perturbations. In the case of larvicide application, a blood fed mosquito can still utilize the pool for oviposition, thus rendering the mosquito ready for another blood meal (and potential secondary inoculations). This result is in line with other recent studies demonstrating the nonlinear effect of targeted larval source management [9,21,25-27]. All of these studies demonstrate the same effect resulting from increased time spent foraging for oviposition habitat. In the absence of the pool, the mosquito must fly greater distance to one of the more peripheral pools in order to oviposit before she can restart her quest for a blood meal. Seeking suitable habitat takes time, and the number of blood meals per mosquito is thus reduced when the pool is drained. This reduction in the number of blood meals per mosquito resulting from spatial factors is reflected in the vectorial capacity for these scenarios. The impacts of treatment and draining of pools 2–4 is not as pronounced, with little or no change in village transmission noted for alterations to pool 4. Pool 4 is the largest, but most distant, pool in the village environment. Some larvae were collected in the field from Pool 4, but the larval abundance was lower than in pools 1 and 2.A further utility of the agent-based model’s ability to track an individual’s source developmental habitat has been shown in Figure 5. Following the 2005 baseline simulation, the parous rates of all simulated mosquitoes were analyzed, and these were mapped to their site of eclosion as shown in Figure 5. Figure 5 shows that the most mosquitoes with parity > 1 (i.e. the ones potentially contributing to malaria transmission) eclosed from pools proximal to the human habitation. The figure illustrates the ability of an agent-based simulation to identify “dangerous” pools. For example, an observer in the field may dip for larval samples in pool 3 and find a high abundance, as was noted in 2005 in Banizoumbou and shown in the model output. However, the mosquitoes destined to eclose from that pool may not play a significant role in village malaria transmission, even though the larval abundance is nearly as high as in pool 1. Although the fates of emergent mosquitoes from individual pools were not determined in the field, the modeling results suggest that not many high-parity mosquitoes emerged from Pool 3 (Figure 5). Pool 1 would be a better use of resources for environmental management, and targeting that pool would have greater impact for vectorial capacity reduction, even though a larval survey would identify comparable aquatic-stage abundances in both pools.

Several other questions may be readily addressed using the agent-based model structure. For instance, mosquito age is tracked by the agent-based model and the model therefore allows the evaluation of age-dependent mortality formulation. Mosquito senescence is significantly underappreciated in malaria models, presumably due to the difficulty of simulation of this process in compartmental models. The potential epidemiological importance of senescence is known, yet rarely modeled [28-31]. Further research will gather field observations of location and parous rate in order to adequately simulate senescence using this model. Mortality can also depend on temperature and humidity experienced by individual mosquitoes, both of which vary seasonally as well as on small scales in microclimates [32]. The model structure can also be exploited for the simulation of immunity resulting from host-vector interactions. The representation of individual humans and mosquitoes allows acquired immunity to result from inoculations allowing simulation of spatial and temporal patterns of host immunity resulting from environmental variability [33].

Further exploitation of the agent-based model’s attribute tracking can be made for the simulation of mosquitoes around Banizoumbou. For example, nutrient enhanced longevity may play a strong epidemiological role. The spatial variability in nutrient content of pools (primarily wind-blown pollen) can be influenced by nearby agricultural activity [34,35]. High levels of wind-blown pollen can settle on the surface of water bodies, which becomes available to the maturing mosquito larvae. The literature presents conflicting results regarding the link between larval nutrition, body size and vectorial capacity [34,36]. However, there is sufficient evidence that well-nourished mosquitoes may be larger and live longer and are thus capable of causing more secondary cases of malaria to warrant exploration of the effect in a model. Other potential model applications include evaluation of the spatial variability of insecticide treated bednet use and indoor residual spraying programs. Because the agent-based model simulates the interactions of individual mosquitoes with humans in the model, when the mosquito is in the host-seeking phase of its life cycle, the mosquito may be killed upon encountering a sleeping human to simulate the effects of an insecticide treated bed net. Moreover, the mosquito may be killed from sprayed walls during its resting phase following a blood meal. It may be that spatial patterns of these interventions yield variable impacts depending on proximity to larval habitat or population density, and such an investigation would also be well suited for an agent-based model such as HYDREMATS. Future research will address these questions.

## Conclusions

Several hypothetical modeling scenarios of a previously-studied transmission environment (Banizoumbou, Niger) have been carried out in order to illustrate the utility of agent-based models for village-scale malaria simulation. The impact of source reduction from pools at various distances was evaluated, not for a quantified estimate of actual impact, but to illustrate the importance of spatial patterns of developmental habitat, proximity to human habitation, and the benefits of agent-based simulation for malaria modeling. Significant differences in simulated vectorial capacity are noted as a result of eliminating different pools at various distances from human habitation, underscoring the importance of spatially-explicit simulation at the village scale. Agent-based modeling can yield significant insight into village-scale malaria transmission that is often neglected in other model paradigms.

## Competing interests

The author declares that he has no competing interests.

## Authors’ contributions

AB conceived of the study, carried out the computer modeling work, analyzed the results, wrote the manuscript and edited the manuscript during the peer review process.
